# MiR-106b exhibits an anti-angiogenic function by inhibiting STAT3 expression in endothelial cells

**DOI:** 10.1186/s12944-016-0216-5

**Published:** 2016-03-09

**Authors:** Ailifeire Maimaiti, Aikebaier Maimaiti, Yining Yang, Yitong Ma

**Affiliations:** The Heart Center, the First Affiliated Hospital of Xinjiang Medical University, No.137 Liyushan South Road, Urumqi, Xinjiang China

**Keywords:** Angiogenesis, Atherosclerosis, Endothelial cells, Micrornas

## Abstract

**Background:**

Recent discoveries of the atherosclerosis-related miRNAs shed new light on the treatment of cardiovascular diseases. Of note, miR-106b ~ 25 cluster and miR-17 ~ 92 cluster are paralogs. Up till now, plenty of researches have shown the role of miR-17 ~ 92 cluster in tumor and atherosclerosis, but miR-106b ~ 25 cluster has stayed mysterious in atherosclerosis field. This study was designed to investigate how miR-106b functions in the atherosclerosis-related angiogenesis and to explore the functioning processes of miR-106b, so as to seek out a new target for the treatment of atherosclerosis.

**Methods:**

Up and down regulation of miR-106b expression was achieved through transfection in HUVECs so as to investigate the function of miR-106b. Next we predicted the target genes of miR-106b and detected them using qRT-PCR and Western blot technique. At last, luciferase assay was conducted to verify the direct target gene of miR-106b. Data are expressed as mean ± SEM. Two treatment groups were compared by Mann–Whitney *U* test or student’s *t*-test. Results were considered statistically significant when *P* < 0.05.

**Results:**

The results showed miR-106b up-regulation groups formed less tubes than control groups while the down-regulation groups showed the opposite. Meanwhile, no obvious effect on apoptosis was observed in endothelial cells. Next we predicted the target genes of miR-106b and finally settled down to *MAPK14* (Mitogen-Activated Protein Kinase)*, STAT3* (Signal Transducers and Activators of Transcription 3), *JAK1*(Janus Kinase 1) and *VEGFA*(Vascular Endothelial Growth Factor A) as candidate target genes. Our results revealed over-expressed miR-106b represses *STAT3* expression, while miR-106b inhibition resulted in *STAT3* up-regulation. Ultimately, luciferase assay confirmed *STAT3* mRNA is the direct target of miR-106b.

**Conclusions:**

Our research demonstrated that miR-106b modulate angiogenesis in endothelial cells through affecting expression of *STAT3*, which occurs by direct target action. Therefore, we affirmed that miR-106b exerts an anti-angiogenic effect in endothelial cells via STAT3-involved signaling pathway, via directly targeting *STAT3.*

## Background

Vascular generation also known as neovascularization refers to vasculogenesis, angiogegnesis and arteriogenesis [1, 2]. In this study, we focused mainly on the angiogenesis process_._Angiogenesis is the process of generating new capillary blood vessels. Physiologically, angiogenesis could be found in wound healing, infections, female reproductive system and also embryogenesis [2, 3]. Unregulated angiogenesis may result in different pathologies such as atherosclerosis, diabetic retinopathy, rheumatoid arthritis and tumor etc [2]. Many atherosclerotic lesions are vascularized by a network of capillaries that arise from the adventitial vasa vasorum. These capillaries may be important regulators of plaque instability. Reflecting their inflammatory microenvironment, the capillaries are immature endothelial tubes with disorganized branching, fragile and prone to rupture. The accumulation of erythrocytes after intraplaque hemorrhage which contains phospholipids and free cholesterol may promote stable plaque to transform into unstable lesion, increasing the probability of cardiovascular events [4]. Studies have shown that the proliferation rates of endothelial cells in plaque capillaries range from undetectable to 43 %, which indicates these vessels are found in various stages of development [5, 6], revealing the key role of endothelial cells in the whole process. Other studies showed that long-term treatment with recombinant murine endostatin or TNP-470 significantly reduced the further growth of atherosclerosis without affecting cholesterol levels [6], which strongly support that intimal capillaries contribute to the progression of atherosclerosis.

Recent discoveries of the atherosclerosis-related miRNAs provoked some thoughts about the diagnosis and treatment of cardiovascular diseases [7]. MiRNAs are a large class of evolutionarily conserved, small, non-coding RNAs, typically 22 nucleotides in length, which primarily function post-transcriptionally by interacting with the 3′untranslated region (UTR) of specific target mRNAs in a sequence-specific manner. MiRNAs have emerged as central regulators of many cardiogenic processes [8]. In the whole history of miRNAs studying, miR-17 ~ 92 cluster was extremely famous for its angiogenesis-related features, no matter in tumors or in atherosclerotic diseases. As its paralog, miR-106b ~ 25 cluster’s role in atherosclerosis still remain to be elucidated. Accordingly, our study mainly focused on how miR-106b act on its target in the angiogenesis processes in endothelial cells.

## Methods

### Cell culture

Human umbilical vein endothelial cells (HUVECs) were isolated from donated umbilical cords and cultivated in ECM medium (Sciencell,Cat.No. 1001) supplemented with ECGs (Sciencell, Cat. No. 1052) plus 5 % FBS (Cat. No. 0025), and used until passage seven. 293 T cells were obtained from Guangzhou RiboBio.Co.Ltd and cultured under the recommended conditions.

### Transfection

For transfection, HUVECs were cultured to 70 % confluence and transfected with 30nM miR-106b mimic (Ambion, Cat. No.4464066) or miR-106 inhibitor (Ambion, Cat. No.AM17000) oligonucleotides using Lipofectin (Invitrogen, Cat. No.18292-011) according to the manufacturer’s instructions. For miR-106b mimic transfection, there were three different treatment groups prepared, which were: mimic group (HUVECs transfected with miR-106b mimic plus Lipofectin), mock group (HUVECs mixed with only Lipofectin) and negative-control group [HUVECs transfected with miRNA mimic negative control (Ambion, Cat. No.4464058) plus Lipofectin]; similarly, for transfection of miR-106b inhibitor, there were inhibitor group (HUVECs transfected with miR-106b inhibitor plus Lipofectin), mock group (HUVECs mixed with only Lipofectin) and negative-control group [HUVECs transfected with miRNA inhibitor negative control (Ambion, Cat. No.4464076) plus Lipofectin]. For transfection of 293 T cells with *STAT3* plasmids, miR-106b mimic was transfected with Lipofectamine^TM^ 2000 (Invitrogen, Cat. No.11668027) to the plasmid/Lipofectamine^TM^ 2000 transfection mix with the final concentrations of 50nM.

### Tube formation assay

Forty eight hours after transfection, HUVECs 200ul (C = 2 × 10^5^/ml) from different treatment groups were cultured respectively in a 48-well plate (Corning) coated with 150 μl Matrigel Basement Membrane Matrix (BD Biosciences) in each well. The number of formed tubes was quantified after 24 h by counting the cumulative tube number in five random microscopic fields, the average number of each group was calculated and normalized to the average number of negative-control group.

### Flow cytometry analysis

For fixation, HUVECs after transfection were detached with trypsin, fixed in 4 % formaldehyde for 10 min and stored in 80 % ethanol. For permeabilization, cells were incubated in 20 μg/ml proteinase K at room temperature for 5 min and then treated with TdT Labeling Reaction Mixture (MERCK, Cat. No.QIA39-1EACN) at 37 °C for 1–1.5 h in the dark. Resuspend cells in 1 × TBS and analyze cells on a FACS Canto II device (BD).

### RNA isolation and RT-PCR

Total RNA of HUVECs was isolated using Qiazol (Qiagen, Cat. No.79306) according to the manufacturer’s protocol. Subsequently, 1 μg of RNA from each sample was reverse transcribed into cDNA and subjected to conventional PCR. To assess the transfection efficiency in HUVECs transfected with miR-106b mimic or inhibitor, we isolated total RNA using Qiazol 24 h after transfection. RT-PCR was performed using the TaqMan MicroRNA Reverse Transcription Kit (Applied Biosystems, Cat. No.4366596) and primers which are specific for reverse transcription of hsa-miR-106 and RNU44 (one cycle: 30 min at 16 °C, 30 min at 42 °C, 5 min at 85 °C). Real–time PCR was performed using the Taqman MicroRNA Assay (Applied Biosystems, Cat. No.4427975) and Taqman Universal PCR Master Mix II (Applied Biosystems, Cat. No.4440040) with RNU44 as loading control. (One cycle: 10 min at 95 °C, 40 cycles: 15 s at 95 °C, 60 s at 60 °C).

To assess the predicted target genes (*MAPK14, STAT3, VEGFA* and *JAK1*) relative expression, we isolated total RNA as stated above and eliminated DNA with DNase I, Amp Grade (Invitrogen, Cat. No.18068-015). RT-PCR was performed using the High-Capacity cDNA Reverse Transcription Kits (Applied Biosystems, Cat. No. 4368814) (one cycle: 10 min at 25 °C, 120 min at 37 °C, 5 min at 85 °C). Real–time PCR was performed using the Power SYBR Green PCR Master Mix (Applied Biosystems, Cat. No.4367659) and primers specific for amplification of *MAPK14, STAT3, VEGFA* and *JAK1* with *GAPDH* (Glyceraldehyde-3-phosphate dehydrogenase) as a reference gene. (One cycle: 10 min at 95 °C, 40 cycles: 15 s at 95 °C, 60 s at 60 °C). The primers used were as follows: *MAPK14*: forward: 5′-GAGCGTTACCAGAACCTGTCTC-3′, reverse: 5′-AGTAACCGCAGTTCTCTGTAGGT-3′; *STAT3*: forward: 5′-CTTTGAGACCGAGGTGTATCACC-3′, reverse: 5′-GGTCAGCATGTTGTACCACAGG-3′; *VEGFA*: forward: 5′-TTGCCTTGCTGCTCTACCTCCA-3′, reverse: 5′-GATGGCAGTAGCTGCGCTGATA-3′; *JAK1*: forward: 5′-GAGACAGGTCTCCCACAAACAC-3′, reverse: 5′-GTGGTAAGGACATCGCTTTTCCG-3′;*GAPDH*: forward: 5′-GGCCTTCCGTGTTCCTACC-3′, reverse: 5′-CGGCATGTCAGATCCACAAC-3′.

### Western blot analysis

For western blot analysis of STAT3 expression, cultured cells were lysed in RIPA (Radio-Immunoprecipitation Assay) buffer on ice and protein concentration was quantified using the Bradford method. Equal amounts of protein were loaded, subjected to SDS-PAGE (Sodium dodecyl sulfate polyacrylamide gel electrophoresis) and blotted on PVDF (Polyvinylidene Fluoride) transfer membranes. Blots were incubated over night with antibodies directed against human STAT3 or GAPDH as a loading control. Proteins were probed with primary antibodies and then with HRP (horseradish peroxidase)-conjugated secondary antibodies. The enhanced chemiluminescence signal was detected using a densitometry program (ChemiDoc XRS, BioRad). To quantify the protein signal, we subtracted background and normalized the value to GAPDH with ImageLab 4.0.

### Luciferase reporter assay

293 T cells (Guangzhou RioBobio Co Ltd, China) (1.5 × 10^4^ cells/well) were seeded in 96-well plates. The predicted binding sequences on the 3′UTR of *STAT3* were synthetically produced by PCR and transferred into a luciferase reporter vector. 100 ng of *STAT3* 3′-UTR luciferase reporter plasmid (Guangzhou RioBobio Co Ltd, China) and either 50 nM miR-106b mimic oligonucleotide or a non-targeting miRNA mimic control (Negative-Control) (Guangzhou RioBobio Co Ltd, China) were co-transfected. All transfections were performed using the Lipofectamine™ 2000 in OPTI-MEM I Reduced Serum Medium (100 μl/well) for 24 h. The luciferase activities from each well were measured using the Dual Glo Luciferase Assay System (Promega, USA) according to the manufacturer’s instruction.

### Statistical analysis

Data are expressed as mean ± SEM. Two treatment groups were compared by Mann–Whitney *U* test or student’s *t*-test (GraphPad Prism 5.). Results were considered statistically significant when *P* < 0.05.

## Results

### Modulation of miR-106b Expression in Cultured HUVECs

**Fig. 1:**
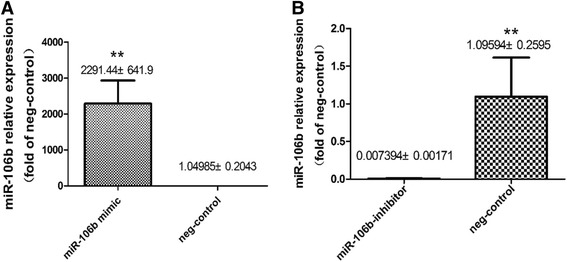
**a**, **b** MiR-106b relative expression in different groups after transfection. Quantitative real-time PCR shows a strong increase in miR-106b in miR-106b mimic transfection group and a dramatic decline in miR-106b levels in miR-106b inhibitor transfection group, which explicitly guaranteed the transfection efficiency. MiR-106b levels were normalized to RNU44, Data are presented as means ± SEM. **P <0.05 (*n* = 4)

To explore the specific role of miR-106b in angiogenesis, we up or down regulated the expression of miR-106b in endothelial cells by using specific miR-106b mimic or inhibitor in transfection, respectively. Mimics and inhibitors with irrelevant nucleotide sequences served as negative controls. We detected transfection efficiency by quantitative real-time PCR which showed a strong increase in miR-106b at 24 h after the mimic transfection, whereas transfection with inhibitor resulted in significantly decreased miR-106b levels (Fig. 1), which precisely confirmed that transfection procedures were successfully accomplished.

### Changes in general function of HUVECs after transfection

**Fig. 2:**
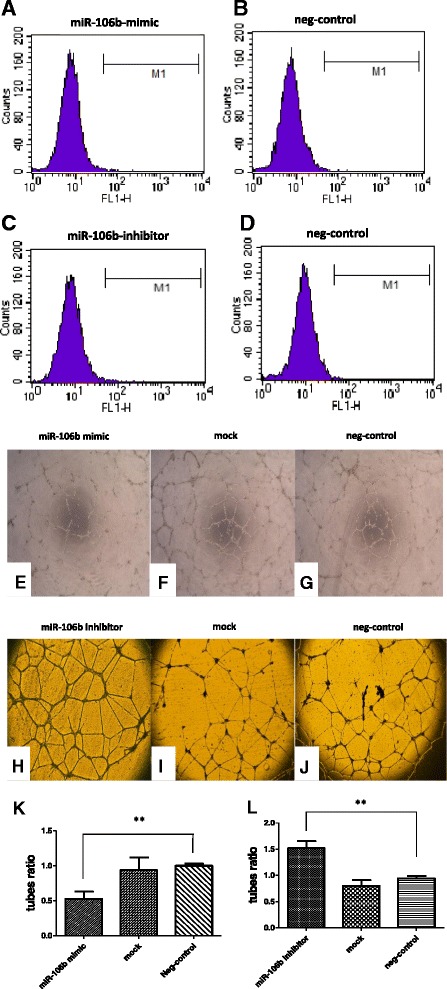
**a**-**l** Flow cytometry analysis and tube formation assay of HUVECs after transfection. **a**-**d** X axis represents fluorescence intensity, while Y axis represents counts of HUVECs. M1 labeled interval stands for the FITC fluorescence intensity range identified by flow cytometry. No matter transfected with miR-106b mimic or miR-106b inhibitor, most of the HUVECs were on the left side of M1 interval, meaning that most of the cells were not apoptotic so they can’t be labeled by fluorescence. **e**-**g** Less tubes were formed in the miR-106b mimic transfection group than the other two groups, furthermore, the discontinuity of tube wall was more significant than the other two groups as well (of 10× magnification). **h**-**j** On the contrary, HUVECs of miR-106b inhibitor transfection group formed more tubes with more intactness than the other two groups (of 25× magnification). **k**, **l** Differences between different treatment groups are significant, data are presented as means ± SEM. **P <0.05, *n* = 4

After transfection, we detected the apoptosis rate of the HUVECs by using TUNEL assay. There was no significant difference in cellular apoptosis after transfection with the different compounds (Fig. 2a-d). The overexpression of miR-106b led to less cell tube formation in the planar Matrigel assay. In contrast, the reduction of miR-106b levels by inhibitor transfection caused increased endothelial tube formation (Fig. 2e-j). Taken together, these findings reveal that miRNA-106b has anti-angiogenic properties in endothelial cells while no obvious effect on apoptosis.

### Screening the targets of miR-106b

Knowing that miR-106b regulates angiogenesis in endothelial cells, we got down to finding out the potential target genes of miR-106b which could be the most key processes involved in the observed effects we mentioned above. A combination of bioinformatic algorithms (Targetscan, MiRanda and PicTar) were used to predict candidate target genes related to angiogenesis, as a result, *VEGFA, JAK1, STAT3, MAPK14, IL-8*(Interleukin-8)*, PDGFRA*(Platelet-Derived Growth Factor Receptor A) were selected.

Through reviewing articles, we summarized several target genes of miR-106b ~ 25 cluster which were validated by other researches (Table 1).

**Table 1:** Currently-known biological functions of miR-106b ~ 25 cluster

microRNAs	Biological function	Target genes	References
miR-93	Preventing the progression of diabetic nephropathy	*VEGF*	[22]
miR-106b ~ 25	Inhibiting cell-cycle arrest and apoptosis	*p21,Bim*	[15, 23]
miR-106b	Impairing cellular cholesterol efflux and increasing the levels of secreted amyloid β	*ABCA1*	[24]
miR-106b,93	Inhibiting brown adipocyte differentiation		[25]
miR-106b ~ 25	Inhibiting apoptosis and promoting tube formation	*PTEN*	[16]
miR-25	Suppressing Cdk inhibitors and promoting cell proliferation	*p57Kip2*	[23]
miR-106b	Inhibiting cell cycle arrest and promoting cell proliferation	*E2F1,E2F3*	[26]
miR-106b	Activating p73 apoptotic signaling in Chronic Lymphocytic Leukemia cells	*ITCH*	[27]
miR-106b	Maintaining the structural homeostasis of developing lung epithelium	*MAPK14*,*STAT3*	[17]
miR-93	Inducing embryonic stem cell differentiation	*STAT3*	[28]

**Fig. 3:**
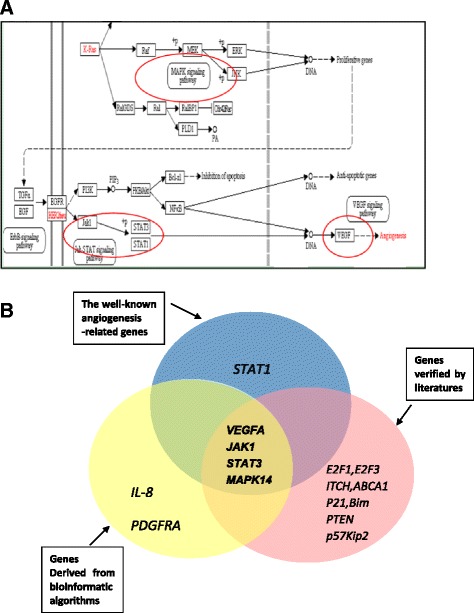
**a**, **b** Signaling pathway obtained from KEGG database and schematic diagram of searching candidate target genes. **a** Among all the pathway, we could easily find the genes associated with angiogenesis, which are, respectively, *MAPK14*, *JAK1*, *STAT1*, *STAT3*, and *VEGF*. **b** We merged genes derived from bioinformatics algorithms, genes proven by literatures and famous angiogenesis-related genes to confirm the final candidate target genes, which are *VEGFA*, *JAK1*, *STAT3* and *MAPK14*

In order to find out how those target genes take part in the angiogenesis processes, we acquired several key signaling pathways involving the candidate target genes by searching currently known signaling pathways related to angiogenesis through Kyoto Encyclopedia of Genes and Genomes (KEGG), the result showed *VEGFA, JAK1, STAT3 and MAPK14* all have irreplaceable roles in angiogenesis (Fig. 3a).

At last, we displayed all the predicted target genes and explored the intersections among them. Then we eventually focused on *VEGFA, JAK1, STAT3* and *MAPK14* (Fig. 3b).

### MiR-106b reduces STAT3 expression in endothelial cells

**Fig. 4:**
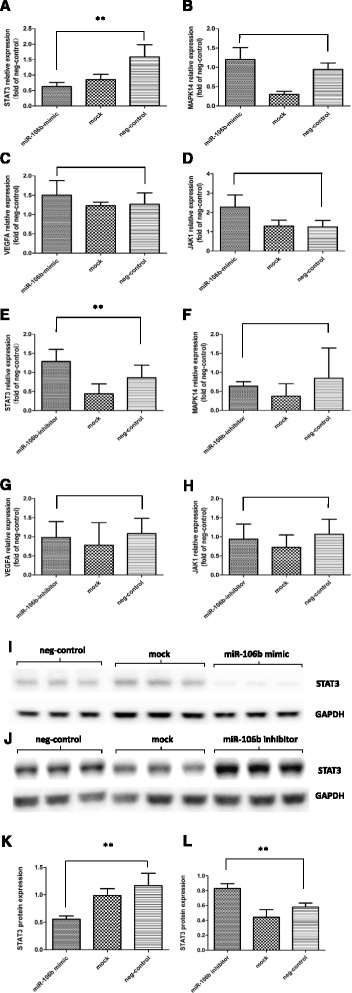
**a**-**l** QRT-PCR results of different candidate genes and western-blot results of STAT3 protein expression among groups under different treatments. **a**-**h** *VEGFA, JAK1* and *MAPK14* expression levels are not significantly affected by miR-106b up or down regulation in HUVECs (*P* >0.05, *n* = 6). Meanwhile, over-expressed miR-106b represses *STAT3* mRNA expression in HUVECs, while miR-106b inhibition lead to an increase in *STAT3* expression. Results are shown as mean ± SEM representative of at least three independent experiments. **P <0.05, *n* = 6. **i-l** Identical to the outcome obtained from qRT-PCR, miR-106b mimic transfection inhibits STAT3 expression while miR-106b inhibitor transfection enhances STAT3 expression. Data are shown as mean ± SEM representative of at least three independent experiments. **P <0.05, *n* = 6

Given that miRNAs function mainly by mRNA repression, we were particularly interested in down-regulated mRNAs because they may be direct miRNA targets. Analysis performed by qRT-PCR revealed that *VEGFA, JAK1* and *MAPK14* expression was unaffected by miR-106b up or down regulation in HUVECs while *STAT3* was regulated by miR-106b as revealed by qRT-PCR and also by Western blot. Our study manifested that overexpressed miR-106b represses *STAT3* expression not only on mRNA but also on protein levels in endothelial cells, while miR-106b inhibition give rise to *STAT3* up-regulation (Fig. 4).

### MiR-106b interfere STAT3′s expression by direct target impact in endothelial cells

**Fig. 5:**
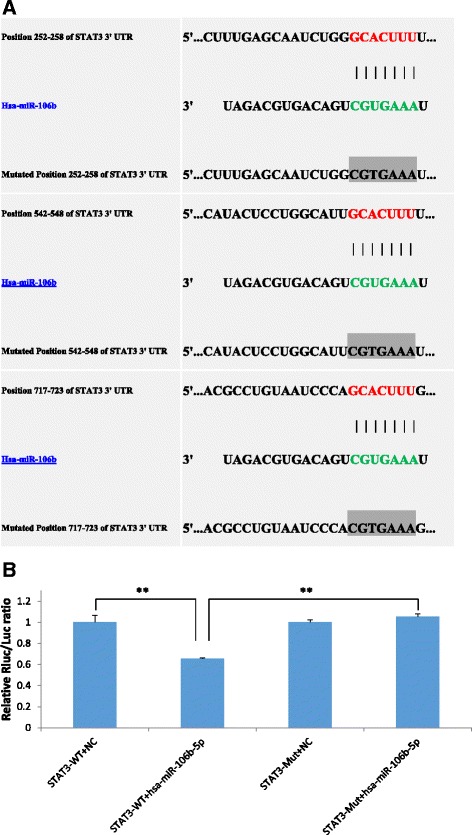
**a**, **b** Luciferase assays of miR-106b binding sites on human *STAT3* 3′UTR. **a** Schematic representation of the 3′ untranslated region (3′UTR) of *STAT3* mRNA with the predicted target sites for miR-106b. The seed sequences of miR-106b are in green letters, the target sites of *STAT3* mRNA are in red letters, and the mutated miR-106b-binding sites are in gray boxes. **b** HUVECs were transfected with either wild-type 3′UTR *STAT3* or mutant 3′UTR *STAT3*, along with the miR-106b mimic. hRluc (renilla luciferace gene) was reporter gene, while hluc (firefly luciferace gene) was reference gene as internal control. Results are shown as mean ± SEM representative of three independent experiments, there are significant differences in fluorescence intensity between *STAT3*-WT + NC transfection group and *STAT3*-WT + miR-106b mimic transfection group and also between *STAT3*-WT + miR-106b mimic transfection group and *STAT3*-MUT + miR-106b mimic transfection group.** *P* < 0.05, *n* = 3

To identify whether *STAT3* is a direct target of miR-106b, we constructed a luciferase reporter vector encoding the complete 3′UTR of *STAT3* [WT (Wildtype) *STAT3* 3′UTR] as well as a control vector containing mismatches in predicted miR-106b binding site [Mut (Mutant) *STAT3* 3′UTR] (Fig. 5a). Co-transfection of the WT *STAT3* 3′UTR plasmid and miR-106b mimic in 293 T cells showed obvious decrease in fluorescence intensity whereas Mut *STAT3* 3′UTR didn’t change luciferase gene expression, which validated that *STAT3* mRNA is a direct target of miR-106b (Fig. 5b).

## Discussion

Notably, miR-106b ~ 25 cluster, miR-17 ~ 92 cluster and miR-106a ~ 363 cluster are paralogs [9],which are located on different chromosomes, derived from a unique gene that underwent a series of duplications, mutations, and loss of individual miRNAs during the early evolution of vertebrates, resulting in the selection of similar but not identical clusters. Based on their seed sequences which are the regions considered most important for target selection (nucleotides 2–7), the miRNAs of these clusters can be grouped into four families: the miR-17 family (miR-17, miR-20a/b, miR-106a/b, and miR-93); the miR-18 family (miR-18a/b); the miR-19 family (miR-19a/b); and the miR-25 family (miR-25, miR-92a, and miR-363). Up till now, plenty of researches have revealed us a profile of up-regulated miR-17 ~ 92 cluster in tumors [10–12], which could promote angiogenesis [13]. Exceptionally, miR-92a has been proven to inhibit angiogenesis in vitro and in vivo [14]. Petrocca et al. [15] discovered that the miR-106b ~ 25 cluster could be activated by E2F1 in a subset of human gastric tumors. In turn, miR-106b and miR-93 regulated *E2F1* expression. Eventually, up-regulation of these miRNAs impaired the TGFβ tumor suppressor pathway through interfering with the expression of *CDKN1A* (p21^Waf1/Cip1^) and *BCL2L11* (Bim). Interestingly, miR-17 ~ 92 cluster synergistically participates in this process. As we previously stated, miR-17 ~ 92 cluster has been proven pivotal in angiogenesis, given the similar biological functions of these two clusters, miR-106b ~ 25 cluster could probably be involved in angiogenesis as well. This idea was recently confirmed by Semo et al [16], who discovered that post-ischemic vascularization in miR-106b ~ 25 knockout mice models of hindlimb ischemia is impaired and subsequent re-expression of miR-106b ~ 25 via local injection of plasmids encoding this cluster can partially reverse the blood supplies. Meanwhile miR-106b ~ 25 knockout bone marrow stromal cells(BMSCs) shows the susceptibilities to apoptosis and decreasing abilities of paracrine and tube formation. All these suggest a pro-angiogenic effect of miR-106b ~ 25 cluster in BMSCs, but the conclusions about miR-106b ~ 25’s pro-angiogenic role in endothelial cells seems not that persuasive, since over-expression of miR-106b ~ 25 in H5V cells has no effect on the tube formation in vitro Matrigel assay. Therefore, to date, the specific role of miR-106b ~ 25 related to angiogenesis in endothelial cells is still obscure. In this study we investigated miR-106b’s role in angiogenesis through observing changes in general functions of endothelial cells after up and down regulating miR-106b’s expression, which demonstrates that miR-106b over-expression in endothelial cells inhibits tube formation while the reduction of miR-106b’s level shows the opposite. No significant difference in TUNEL assay suggests that miR-106b’s expression has no obvious effect on apoptosis in endothelial cells.

Knowing that miR-106b modulates angiogenesis in endothelial cells, we wondered how these processes could be achieved. Since miRNAs function through inhibiting expression of their target genes, seeking out the potential target genes of miR-106b would explain the pivotal mechanisms giving rise to the observed effects. Angiogenesis-related target genes were screened out via a combination of bioinformatic algorithms (Targetscan, MiRanda and PicTar), consequently, *VEGFA, JAK1, STAT3, MAPK14, IL-8, PDGFRA* were selected to be the candidate genes. With the purpose of choosing the most likely genes, we consulted a certain amount of literatures to search currently known target genes associated with miR-106b ~ 25 cluster. As a member of miR-17 family, miR-106b shares an identical seed region with other family members. Therefore, it can be inferred that they may share a number of target genes. Studies showed miR-17 family contributes to maintain the structure stability of alveolar epithelial cells in growth and development process by targeting *MAPK14* and *STAT3* [17], meanwhile other evidence demonstrated that miR-17 family function as direct endogenous repressors of the *VEGFA* in nasopharyngeal carcinoma cells [18]. Researches of endothelial cells manifested that miR-17 has an anti-angiogenic function by inhibiting the expression of target gene *JAK1* and subsequent repression of JAK/STAT signaling in endothelial cells [19]. Kyoto Encyclopedia of Genes and Genomes (KEGG) provided us with several angiogenesis-associated signaling pathways involving *VEGFA, JAK1, STAT1, STAT3* and *MAPK14*, which are turn out to be crucial in angiogenesis processes.

Taken together, we speculate that *MAPK14, STAT3, VEGFA and JAK1* could be the potential target genes of miR-106b. Based on this speculation, our study focused on how miR-106b act on its target in endothelial cell-related angiogenesis. Data gathered from the qRT-PCR and Western blot demonstrated that up or down regulation of miR-106b in HUVECs has no influence on expression of *VEGFA, JAK1* and *MAPK14*, while expression of *STAT3* would be repressed by miR-106b. Therefore, there is every reason to suspect that miR-106b inhibits *STAT3* expression via directly binding to it. Consistent with Carraro G et al [17], luciferase reporter assay showed obvious decrease in fluorescence intensity when WT *STAT3* 3′UTR plasmid and miR-106b mimic co-transfected together whereas Mut *STAT3* 3′UTR didn’t change luciferase gene expression, which verified that miR-106b inhibits *STAT3* expression by a direct target effect. Thus, our research explicitly revealed miR-106b suppresses *STAT3* expression, which due to a direct effect mediated by miR-106b, that is to say, miR-106b exhibits an anti-angiogenic function by inhibiting *STAT3* expression in endothelial cells.

In this research, we have confirmed miR-106b directly inhibited *STAT3* expression with no obvious effect on *MAPK14*, *VEGFA* and *JAK1,* which was partially consistent with Carraro’s [17] discovery that miR-17 family downregulate *STAT3* and *MAPK14* expression in lung epithelial cells. As is known to all, STAT3 pathway was identified as key components linking cytokine signals to transcriptional events in cells, meanwhile STAT proteins can regulate many pathways including cell-cycle progression, apoptosis, tumor angiogenesis, tumor-cell invasion and metastasis, and tumor-cell evasion of the immune system [20]. Niu et al [21] demonstrated that VEGF expression correlated with STAT3 activity in various human cancer cell lines. An activated STAT3 mutant upregulated VEGF expression and promoted tumor angiogenesis. Moreover, interrupting STAT3 signaling in tumor cells downregulated VEGF expression and inhibited angiogenesis. Apparently, STAT3 pathway promote angiogenesis primarily through upregulating VEGFA expression, however, our results showed that VEGFA level was not affected. We considered STAT3 may regulate other angiogenic factors, such as HIF-1α and FGF2, to promote angiogenesis process. Originally, our research was designed to find out miR-106b’s function in endothelial cells based on the predicted target genes of miR-106b through TargetScan/MiRanda/PicTar algorithms, according to which, *HIF-1α* and *FGF2* are not predicted target genes of miR-106b. Therefore, detecting those factors does not conform to our original research intention. Nevertheless, in our subsequent research plan, we are focusing on this issue to ensure its completion.

## Conclusions

Our research found that miR-106b restrains angiogenesis processes in endothelial cells, meanwhile expression of *STAT3* is significantly repressed, which was subsequently proven to be a direct effect between miR-106b and *STAT3*. Given the importance of STAT3 in angiogenesis, we conclude that miR-106b exhibits an anti-angiogenic effect in endothelial cells through directly binding to *STAT3*.

### Ethics approval and consent to participate

Not applicable.

### Consent for publication

Not applicable.

## Availability of data and materials

The raw data cannot be shared for now, since the data may be used in our subsequent researches.

## Abbreviations

GAPDH: Glyceraldehyde-3-phosphate dehydrogenase
IL-8: Interleukin-8
JAK1: Janus kinase 1
KEGG: Kyoto encyclopedia of genes and genomes
MAPK14: Mitogen-activated protein kinase
Mut: Mutant
PDGFRA: Platelet-derived growth factor receptor A
PVDF: Polyvinylidene fluoride
RIPA: Radio-immunoprecipitation assay
SDS-PAGE: Sodium dodecyl sulfate polyacrylamide gel electrophoresis
STAT3: Signal transducers and activators of transcription 3
VEGFA: Vascular endothelial growth factor A
WT: Wildtype
